# Differences in mental indicators and state-hope related to the level of engagement in social unrest: Israel 2023

**DOI:** 10.3389/fpubh.2023.1284211

**Published:** 2024-01-05

**Authors:** Yaira Hamama-Raz, Elazar Leshem, Menachem Ben-Ezra

**Affiliations:** School of Social Work, Ariel University, Ariel, Israel

**Keywords:** anxiety, distress, engagement in social unrest, mental health, state-hope

## Abstract

**Background:**

This study examined the interplay between engagement in social unrest, mental indicators, state-hope and demographic variables. In addition, mental indicators and state-hope were compared in line with levels of engagement in social unrest.

**Methods:**

In a cross-sectional study, conducted from March 23 to April 10, 2023, 2031 Israelis were recruited via a survey company. Participants completed self-report questionnaires to assess engagement in social unrest, anxiety, social unrest related distress, state-hope and demographic variables.

**Results:**

Participants with higher engagement in social unrest, who opposed the law reform, were prone to higher levels of social unrest related distress, anxiety, and lower levels of state-hope compared to those not engaged in social unrest activities or those who supported the law reform.

**Conclusions:**

Concerns regarding unmet mental health needs, during and following social unrest, regardless of the engagement level, should be actively addressed by mental health professionals and health policy makers.

## Differences in mental indicators and state-hope related to the level of engagement in social unrest: Israel 2023

Social unrest is a major social issue in countries with advanced economies as well as in countries with emerging and developing economies throughout the world (1). Social unrest is rising globally (2), and its impact on the mental health of the world's population should be assessed (2). In this context, Ni and colleagues (3) conducted the first systematic review of collective actions and effects on mental health and found that civil protests even when nonviolent can be associated with adverse mental health outcomes, namely post-traumatic stress disorder (ranging from 4% to 41% in riot-affected areas), major depression (increased by 7%, regardless of personal involvement in the protests), and anxiety (ranging between 10.5% immediately after the event to 47.4% 2 months later showing moderate-to-severe anxiety) (3). Moreover, after the 2019 anti-ELAB movement in Hong Kong Tao et al. (4) investigated widespread civil unrest in Hong Kong between July 2019 and July 2020 and revealed that unrest-related distress was positively associated with probable depression across different numbers of conflicts/protests.

In contrast, several previous studies of social unrest (5) found that collective actions were associated with improved mental health—reduced depression and suicide and was found to protect against psychological distress (6). It is possible that engagement in collective action provides greater social cohesion among activists, which in turn could buffer the adverse impact of stressful situations that may accompany engagement in social unrest. In line with this context Gorski (7) found via a phenomenological study among racial justice activists in the United States, that engaging in collective action also resulted in feelings of empowerment and social support.

With regard to the associations of demographic factors, engagement with social unrest and mental health outcomes, anxiety and depressive symptoms correlated with younger age, female sex, lower education level, and lower socioeconomic status (3, 8). In addition, unemployment was associated with more depressive symptoms during and after being engaged in civil unrest (9).

Several scholars stressed that positive mental health can be promoted through psychological factors including hope (10, 11). Specifically, hope was associated with a positive affect and higher flourishing, greater life satisfaction, enhanced perceptions that life is meaningful, and a higher sense of purpose in life (11–13). Conversely, low levels of hope have been positively associated with elevated risk of mental health problems such as anxiety, depression, and posttraumatic stress disorder (14). Nevertheless, hope is thought to have positive value in the political sphere as it makes desirable political outcomes more likely (15). In this context, hope was found to facilitate not only normative but also non-normative collective action (16). Indeed, several studies highlighted the role of hope in mobilizing people to act for change (17–19). Cohen-Chen and Van Zomeren (20) revealed that hope acts as a moderator on the efficacy-based pathway to collective action, such that perceived efficacy predicts collective action only among individuals who maintain high hopes for social change.

Given the above, the present study aimed to assess the mental health toll (namely anxiety, and distress related to social unrest) as well as state-hope among the Israeli population which has currently been experiencing social unrest, as the impact of these factors is currently unknown in the Israeli population.

Indeed, Israel recently experienced an outburst of civil unrest. A series of street protests, strikes and hunger strikes began in early 2023 in response to the ruling government's push for a broad judicial reform. The protests have been taking place in cities across the country every Saturday since January 7^th^, as well as on other days of the week from time to time (21). Over 630,000 people who opposed the judicial changes have attended the protests (22). However, protests supporting the judicial reform also began in response to the anti-reform protests. These additional protests intensified social unrest and even gave rise to fears of civil war.

Thus, the aims of this study were threefold: First, to assess associations between engagement in social unrest, demographic variables, mental indicators and state-hope; Second, to compare mental indicators and state-hope between those who participate in social unrest and those who do not; Third, to reveal the predictors of mental indicators and state-hope. We hypothesized that: (1) Engagement in social unrest will be associated with (a) higher anxiety and, higher distress related to social unrest; (b) higher state-hope. (2) People who participate in the present social unrest will report higher anxiety, higher social unrest related distress and higher state-hope in comparison to their counterparts who do not participate in the protests. (3) Mental indicators and state-hope will be predicated by demographic variables and engagement in social unrest. The highest contribution to the explained variance will be related to engagement in social unrest.

## Methods

### Recruitment and eligibility

Data were collected from March 23 to April 10, 2023. Eligibility criteria were age 18 or over; Israeli residents at the time the survey was conducted; able to give informed consent; fluent in Hebrew—the native language.

### Sample size

We estimated that at least 1895 participants would be required to detect low effect sizes of 0.02, with 99% power and at 1% significance level based on the inclusion of 8 predictor variables, in a linear regression model.

### Sampling and procedures

The study was conducted according to the STROBE guidelines for observational studies. We used Israel's *iPanel* company to conduct the survey. This probability-based panel has over 100,000 members (23). The panels consist of adults aged 18–85 who have given their consent to be contacted for surveys. Panel recruitment is dynamic and ongoing, using a range of online methods. *iPanel* adheres to the stringent standards of the world association for market, social, and opinion researchers (ESOMAR).

A quota sampling approach was used with quotas meeting the Israeli national census data for age and sex, as specified by the Israeli Bureau of Statistics census data. The use of this approach ensured a good representation of the adult population in Israel. After the quotas and required sample size were reached, the survey was closed.

The final data set was weighted according to these factors (age and sex) to enable the study to be considered representative of internet-using participants ages 18–85 years living in Israel.

The sample was recruited online, and all participants signed an electronic informed consent form. The study was approved by the first author's Institutional Review Board. Out of 3,500 invitations sent, 2,421 responded fully and 390 were excluded due to over quota. Final sample size was 2,031 (Response Rate = 69.17%).

Of the 2,031 participants, 50.4% of the sample (*n* = 1,024) were men and 49.6% (*n* = 1,007) were women, aged 18–85 years (*M* = 42.54, *SD* = 15.66). Most of the participants were in a committed relationship (57.9% of the sample, *n* = 1,176). Regarding religion, 1,108 (54.6%) reported being secular, 585 (28.8%) traditional and 338 (16.6%) religious. The mean years of education was 14.43 (*SD* = 2.74). Income level shows that 502 participants (24.7%) report income below the monthly average 12,471 NIS [3,396 USD/3,059 EUR/2,734 GBP], 1,118 participants (55.0%), reported average income, 411 participants (20.2%) reported an above average income.

### Measurements

All participants completed several self-report measures, as follows:

Demographic information: Participants provided details about their age, biological sex, relationship status, years of education, economic status, and religiosity level. Self-rated health was measured with the question: “In general, how do you rate your health?” rated on a four-point Likert scale ranging from “1” excellent to “4” bad. Five hundred fifty-two participants rated their health as excellent (27.5%), 1,239 as good (61.6%), 200 as not so good (10.0%) and 19 as bad (0.9%). This single-item measure was found to be valid and highly associated with objective indicators of health (24).

*Social unrest engagement* related to the law reform was measured with four questions divided to two questions opposing the law reform namely (1). “Did you sign petitions opposing the law reform?” and (2). “Did you attend civil demonstrations opposing the law reform.” “0” coded as “no” and “1” coded as “yes.” There were also two questions regarding support of the law reform namely, (1). “Did you sign petitions in support of the law reform?” and (2). “Did you attend civil demonstrations in support of the law reform.” “0” coded as “no” and “1” coded as “yes” (Cronbach's α = 0.66). We combined the questions to create an index of civil unrest engagement opposing/supporting the law reform. The combined index has five groups and ranged from 1 to 5. The coding designated “1” as high social unrest engagement opposing the law reform, “2” low social unrest engagement opposing the law reform, “3” not engaged, “4” low social engagement supporting the law reform, and “5” high social engagement supporting the law reform.

*Social Unrest Distress* was measured with four questions based on Hou and colleagues' (25) assessment of unrest distress in Hong Kong and modified to address Israel reality. That is: (1). “I felt distressed regarding how the government handled the protest against the law reform.” (2). “I felt distressed regarding the clashes between the police and the protestors and the use of riot control measures.” (3). “I felt distressed regarding the social unrest, protests, and demonstrations against the law reform and how they disrupt the daily routine.” (4). “I felt distressed regarding the repercussions of the social unrest on the social, economic and security conditions in Israel.” Each item was rated on a four-point Likert scale ranging from “1” not at all to “4” very much. These questions were summed in order to create a civil unrest distress index. The internal consistency of this index was adequate (Cronbach's α = 0.84).

*Anxiety* was measured via the International Anxiety Questionnaire (IAQ); (26), a self-report measure of ICD-11 Generalized Anxiety Disorder (ICD-11 diagnostic code 6B00). It can be used to generate severity scores and to identify cases meeting diagnostic criteria. The severity scoring method simply involves summing the scores of the 8 IAQ items producing a possible range of scores from 0 to 32. Each item is rated on a five-point Likert scale from “0” Never to “4” every day” (e.g., “Felt your heart racing, difficulty breathing, stomach discomfort, or dry mouth?”). The internal consistency of the IAQ was good (Cronbach's α = 0.93).

*State- hope* was measured using the State Hope Scale (SHS); (27). This six-item scale includes three agency and three pathway statements related to how respondents perceive themselves “right now.” Each item is rated on an eight-point Likert scale ranging from “1” definitely false to “8” definitely true. Summation of the items 1,3,5 (score range from 3–24) compose the pathways subscale (e.g., “I can think of many ways to reach my current goals”), and summation of items 2,4,6, (score range from 3–24) compose the agency subscale score (e.g., “At the present time, I am energetically pursuing my goals”). The total hope score is the summation of the two subscales. Scores can range from 6 to 48, with higher scores representing higher hope levels. The internal consistency of the SHS scale was good (Cronbach's α = 0.90).

### Statistical analysis

First, we conducted a basic correlation matrix of the study variables. Second, we conducted a preliminary ANOVA analysis with Scheffe *post-hoc* tests (engagement in social unrest as the independent variable and social unrest related distress, ICD-11 anxiety and state hope as the dependent variable) followed by a MANCOVA with age, biological sex, relationship status, religiosity, years of education, economic status, and self-rated health as covariates. The main factor was engagement.in social unrest the dependent variables were social unrest related distress, ICD-11 anxiety, and state hope. Third, we conducted three separate linear regressions with age, biological sex, relationship status, religiosity, years of education, economic status, and self-rated health entered the equation in step one. In step two, we entered engagement, in social unrest. We entered the dependent variables namely social unrest related distress, ICD-11 anxiety, and state hope, respectively.

## Results

The results section is composed of three parts. Correlation matrix, simple ANOVA with *post-hoc* Scheffe test and MANCOVA, and multiple hierarchical regression results, respectively.

Engagement in social unrest correlated with older age (r = −0.133; *p* < 0.001), female sex (r = −0.086; *p* < 0.001), being secular (r = 0.368; *p* < 0.001), higher education (r = −0.160; *p* < 0.001), higher economic status (r = −0.129; *p* < 0.001), experiencing more civil unrest related distress (r = −0.348; *p* < 0.001), being more anxious (r = −0.200; *p* < 0.001). For more information, see Table 1.

**Table 1:** Correlation matrix of the study variables (*n* = 2,031).

	**1**	**2**	**3**	**4**	**5**	**6**	**7**	**8**	**9**	**10**	**11**
1. Age	–										
2. Sex	−0.086^***^	–									
3. Relationship status	0.343^***^	−0.074^***^	–								
4. Religiosity	−0.023	0.005	0.186^***^	–							
5. Year of education	0.204^***^	0.054^**^	0.152^***^	−0.050^**^	–						
6. Economic status	0.163^***^	−0.121^***^	0.274^***^	−0.060^***^	0.264^***^	–					
7. Self-Rated health	0.295^***^	0.008	0.037	−0.061^**^	0.024	−0.123^***^	–				
8. Social unrest engagement	−0.133^***^	−0.045^*^	0.033	0.368^***^	−0.160^***^	−0.129^***^	−0.031	–			
9. Civil unrest distress	0.141^***^	0.166^***^	−0.011	−0.249^***^	0.109^***^	0.022	0.155^***^	−0.348^***^	–		
10. Anxiety	−0.135^***^	0.160^***^	−0.140^***^	−0.144^***^	−0.041	−0.155^***^	0.223^***^	−0.200^***^	0.436^***^	–	
11. State- Hope	0.090^***^	−0.013	0.135^***^	0.053^**^	0.143^***^	0.222^***^	−0.210^***^	0.027	−0.096^***^	−0.311^***^	–

^*^p < 0.05; ^**^p < 0.01; ^***^p < 0.001.

### Differences in mental indicators according to levels of engagement in social unrest

We conducted a preliminary simple ANOVA analysis with engagement in social unrest as the main factor and social unrest related distress, anxiety and state hope as the dependent variables with Scheffe *post-hoc* comparisons. The most significant results were found for social unrest related distress (F = 75.590; *p* < 0.001) and anxiety (F = 23.306; *p* < 0.001) and to a lesser extent state-hope (F = 4.915; *p* < 0.001). The Scheffe *post-hoc* comparisons were found significant among the groups with social unrest related distress where those who opposed the law reform significantly differed from the other groups (1 differs from 3, 1 differs from 4, 1 differs from 5, 2 differs from 3, 2 differs from 4, 2 differs from 5, 3 differs from 4) and similar but less significant among the groups in anxiety where those who opposed the law reform differed less significantly from the other groups (1 differs from 3, 1 differs from 4, 2 differs from 3, 2 differs from 4, 3 differs from 4). The results of the state-hope evaluations were sporadic (2 differs from 4, 3 differs from 4).

Descriptive results of the MANCOVA, suggest that those who are engaged in social unrest (against the law reform), have higher social unrest related distress (Mean = 12.78, SD = 2.51 and Mean = 12.20, SD = 3.08) vs. unengaged and those who support the law reform (Mean = 9.89, SD = 3.63, Mean = 8.43, SD = 3.32 and Mean = 8.74, SD = 3.49) and same for anxiety (Mean = 19.91, SD = 7.31 and Mean = 18.42, SD = 7.53 vs. Mean = 16.55, SD = 6.83, Mean = 13.83, SD = 6.41 and Mean = 16.58, SD = 6.17) in comparison to those who are not engaged or support the reform. To a lesser extent state-hope was found somewhat higher among those who support the reform in comparison to the unengaged and those who oppose the reform (Mean = 35.15, SD = 11.28 and Mean = 35.33, SD = 8.32 vs. Mean = 33.14, SD = 8.00, Mean = 31.76, SD = 8.31 and Mean = 32.35, SD = 8.91). See Table 2, for more information.

**Table 2:** Descriptive statics of the MANCOVA analysis.

	**Political engagement groups**	**Mean**	**Std. Deviation**	**N**
Civil unrest distress	High against the law reform	12.7778	2.50899	297
	Low against the law reform	12.2000	3.08159	290
	Non-engaged	9.8884	3.62978	1,263
	Low in favor of the law reform	8.4309	3.31930	123
	High in favor of the law reform	8.7308	3.49351	26
Anxiety	High against the law reform	19.9057	7.31006	297
	Low against the law reform	18.4241	7.53328	290
	Non-engaged	16.5527	6.83460	1,263
	Low in favor of the law reform	13.8293	6.40531	123
	High in favor of the law reform	16.5769	6.16554	26
State hope	High against the law reform	33.1448	7.99678	297
	Low against the law reform	31.7552	8.31341	290
	Non-engaged	32.3523	8.90877	1,263
	Low in favor of the law reform	35.3252	8.31796	123
	High in favor of the law reform	35.1538	11.28075	26

The results of the MANCOVA revealed that engagement in social unrest was a significant factor for social unrest related distress (F = 42.466; *p* < 0.001; partial η^2^ = 0.079), anxiety (F = 24.160; *p* < 0.001; partial η^2^ = 0.046) and state hope (F = 4.152; *p* = 0.002; partial η^2^ = 0.008). In addition, some demographic factors were also found significant such as age for social unrest related distress (F = 9.483; *p* = 0.002; partial η^2^ = 0.005), anxiety (F = 76.301; *p* < 0.001; partial η^2^ = 0.037) and state hope (F = 17.197; *p* < 0.001; partial η^2^ = 0.009), biological sex for social unrest related distress (F = 63.262; *p* < 0.001; partial η^2^ = 0.031), anxiety (F = 35.528; *p* < 0.001; partial η^2^ = 0.018), religiosity for social unrest related distress (F = 37.117; *p* < 0.001; partial η^2^ = 0.018), anxiety (F = 6.884; *p* = 0.009; partial η^2^ = 0.003), years of education for state hope (F = 15.466; *p* < 0.001; partial η^2^ = 0.008), economic status for anxiety (F = 18.680; *p* < 0.001; partial η^2^ = 0.009) and state hope (F = 42.239; *p* < 0.001; partial η^2^ = 0.021). Finally, self-rated health for social unrest related distress (F = 28.292; *p* < 0.001; partial η^2^ = 0.014), anxiety (F = 144.833; *p* < 0.001; partial η^2^ = 0.068) and state hope (F = 93.648; *p* < 0.001; partial η^2^ = 0.045). For more information, see Table 3.

**Table 3:** MANCOVA results of the study variables.

**Source**	**Dependent variable**	**Type III sum of squares**	**Mean square**	**F**	**Sig**.	**Partial Eta squared**
Age	Social unrest related distress	101.000	101.000	9.483	0.002	0.005
	Anxiety	3,209.413	3,209.413	76.301	< 0.001	0.037
	State hope	1,163.885	1,163.885	17.197	< 0.001	0.009
Biological sex	Social unrest related distress	673.777	673.777	63.262	< 0.001	0.031
	Anxiety	1,494.396	1,494.396	35.528	< 0.001	0.018
	State hope	68.968	68.968	1.019	0.313	0.001
Relationship status	Social unrest related distress	1.806	1.806	0.170	0.681	0.000
	Anxiety	31.783	31.783	0.756	0.385	0.000
	State hope	309.019	309.019	4.566	0.033	0.002
Religiosity	Social unrest related distress	395.315	395.315	37.117	< 0.001	0.018
	Anxiety	289.575	289.575	6.884	0.009	0.003
	State hope	26.210	26.210	0.387	0.534	0.000
Years of education	Social unrest related distress	17.413	17.413	1.635	0.201	0.001
	Anxiety	42.459	42.459	1.009	0.315	0.001
	State hope	1,046.753	1,046.753	15.466	< 0.001	0.008
Economic status	Social unrest related distress	3.146	3.146	0.295	0.587	0.000
	Anxiety	785.727	785.727	18.680	< 0.001	0.009
	State hope	2,858.765	2,858.765	42.239	< 0.001	0.021
Self-rated health	Social unrest related distress	301.328	301.328	28.292	< 0.001	0.014
	Anxiety	6,092.052	6,092.052	144.833	< 0.001	0.068
	State hope	6,338.089	6,338.089	93.648	< 0.001	0.045
Social unrest engagement	Social unrest related distress	1,809.124	452.281	42.466	< 0.001	0.079
	Anxiety	4,064.961	1,016.240	24.160	< 0.001	0.046
	State hope	1,123.960	280.990	4.152	0.002	0.008

### Predictors of mental indicators

The results of the hierarchical regressions echo the previous results. The regression model for civil unrest related distress was significant in step 1 [R = 0.358; R2 = 0.128; F_(7, 1991)_ = 41.694; *p* < 0.001] and improved in step 2 [R = 0.435; R^2^ = 0.189; F_(1, 1990)_ = 150.868; *p* < 0.001]. The same result was found for anxiety in step 1 [R = 0.371; R^2^ = 0.138; F_(7, 1991)_ = 45.507; *p* < 0.001] and that improved in step 2 [R = 0.416; R2 = 0.173; F_(1, 1990)_ = 85.201; *p* < 0.001] and state-hope in step 1 [R = 0.330; R^2^ = 0.109; F_(7, 1991)_ = 34.686; *p* < 0.001] but showed less improvement in step 2 [R = 0.334; R^2^ = 0.111; F_(1, 1990)_ = 6.227; *p* < 0.013].

Across the social unrest related distress, anxiety, and state-hope, there were common variables that were found to be significant: engagement in social unrest (β ranged from −0.273 to 0.058 with t ranging from −12.283 to 2.495 and *p*-value ranging from < 0.001 to 0.013). A similar pattern was found with age (β ranged from −0.202 to 0.106 with t ranging from −8.641 to 4.373 and *p*-value ranging from < 0.001 to 0.001) and self-rated health (β ranged from −0.221 to.260 with t ranging from −9.800 to 11.966 and *p* values were at < 0.001). To a lesser extent, biological sex was significantly associated with social unrest related distress (β = 0.161; t = 7.856; *p* < 0.001) and anxiety (β = 0.120; t = 5.804; *p* < 0.001), while religiosity had the same pattern with social unrest related distress (β = −0.142; t = −6.371; *p* < 0.001) and anxiety (β = −0.059; t = −2.622; *p* = 0.009) while economic status was found to be significant with anxiety (β = −0.095; t = −4.239; *p* < 0.001) and state-hope (β = 0.154; t = 6.595; *p* < 0.001). Finally, state-hope was found to be significantly associated with relationship status (β = 0.047; t = 1.985; *p* = 0.047) and years of education (β = 0.088; t = 3.929; *p* < 0.001). For more information, see Table 4.

**Table 4:** Hierarchical regression of the study variables.

**Variables**	**Social unrest distress**			**Anxiety**			**State- hope**		
	β	**T**	**p**	β	**T**	**p**	β	**T**	**p**
**Step 1**									
Age	0.106	4.419	< 0.001	−0.179	−7.546	< 0.001	0.100	4.126	< 0.001
Sex	0.177	8.314	< 0.001	0.132	6.248	< 0.001	0.015	0.684	0.494
Relationship status	−0.003	−0.135	0.893	−0.031	−1.313	0.189	0.049	2.074	0.038
Religiosity	−0.237	−10.976	< 0.001	−0.131	−6.114	< 0.001	0.044	1.994	0.046
Years of education	0.061	2.741	0.006	0.002	0.078	0.938	0.083	3.681	< 0.001
Economic status	0.010	0.453	0.651	−0.079	−3.458	< 0.001	0.149	6.412	< 0.001
Self-Rated health	0.109	4.892	< 0.001	0.257	11.586	< 0.001	−0.220	−9.751	< 0.001
**Step 2**					17.428	< 0.001		15.197	< 0.001
Age	0.075	3.251	0.001	−0.202	−8.641	< 0.001	0.106	4.373	< 0.001
Sex	0.161	7.856	< 0.001	0.120	5.804	< 0.001	0.018	0.836	0.403
Relationship status	0.007	0.307	0.759	−0.023	−1.004	0.316	0.047	1.985	0.047
Religiosity	−0.142	−6.371	< 0.001	−0.059	−2.622	0.009	0.023	0.999	0.318
Years of education	0.033	1.530	0.126	−0.019	−0.895	0.371	0.088	3.929	< 0.001
Economic status	−0.011	−0.490	0.624	−0.095	−4.239	< 0.001	0.154	6.595	< 0.001
Self-Rated health	0.113	5.258	< 0.001	0.260	11.966	< 0.001	−0.221	−9.800	< 0.001
Engagement in social unrest	−0.273	−12.283	< 0.001	−0.207	−9.230	< 0.001	0.058	2.495	0.013
**Model summary**									
Social unrest distress			Step 1: R = 0.358; R^2^ = 0.128; F_(7, 1991)_ = 41.694; *p < * 0.001 Step 2: R = 0.435; R^2^ = 0.189; F_(1, 1990)_ = 150.868; *p < * 0.001						
Anxiety			Step 1: R = 0.371; R^2^ = 0.138; F_(7, 1991)_ = 45.507; *p < * 0.001 Step 2: R = 0.416; R^2^ = 0.173; F_(1, 1990)_ = 85.201; *p < * 0.001						
Hope			Step 1: R = 0.330; R^2^ = 0.109; F_(7, 1991)_ = 34.686; *p < * 0.001 Step 2: R = 0.334; R^2^ = 0.111; F_(1, 1990)_ = 6.227; *p < * 0.013						

## Discussion

The social unrest in Israel provides a unique context for revealing differences in mental indicators and state-hope according to the level of engagement in social unrest (those who oppose the law reform compared to their counterparts who support the law reform). Specifically, as hypothesized, our findings show that engagement in the social unrest in Israel (against the law reform) was associated with higher mental indicators (anxiety and social unrest). However, surprisingly, only a small and weak association was found in reference to state-hope. Concerning the mental indicators, our results support previous results which state that civil unrest is related to poor mental health or emotional distress (3, 8). This result might be explained through the notion of disruption to normal routines which accompanies social unrest. Indeed, previous findings indicated the role of normal routines as a protective buffer against mental illnesses (28). Moreover, as the social unrest in Israel also created tension in interpersonal relations among family members, close friends, and colleagues (29), it might be that the interpersonal tension raised psychological distress, anxiety and fear of a possible civil war. In line with this notion, a recent study conducted among older people in Hong Kong following social unrest (30) revealed that participants reported experiencing changes in their relationships with their family members. Some had conflicts with family members, because of different opinions while others intentionally avoided talking about the situation with their family members in order to avoid conflict (30).

Regarding the weak association between engagement in social unrest and state-hope, a possible explanation might stem from the hope hierarchy model (31). At the bottom of the hierarchy there is the facet of hope that is most stable across time and experiences: trait hope. While trait hope involves an individual's self-perception of his or her aptitude for general goal attainment, the next level of the hierarchy, state-hope, involves one's momentary perception of his/her ability for goal attainment. As such, it is less stable across time and experiences than trait hope, fluctuating with and influenced by a person's mood across circumstances and time. Thus, it is possible that during the present study, state-hope played a minor role in engagement in social unrest. However, if the social unrest continues, and additional political changes occur in Israel, findings will be different.

With respect to the differences in mental indicators and state-hope according to level of engagement, our findings revealed an interesting picture. Specifically, anxiety level was higher among those against the law reform with both high and low engagement while the lowest level of anxiety was reported among those in favor of reform with low engagement in social unrest. Surprisingly, participants who reported non-engagement in the social unrest revealed quite similar anxiety levels as their counterparts in favor reform with high engagement in social unrest. These findings suggest that even non engagement in social unrest carries a mental toll, as social unrest can cause “snowball effects” in broad aspects of life including economic, social cohesion and sense of security. Moreover, offline street protests may continue as “online protests” on social media platforms and vice versa (32). A recent study and systematic review revealed that social media may also act as a stressor due to interactions with other online users with different ideological views (3). Thus, even individuals who are not engaged in social unrest might be exposed to the high complexity, uncertainty, and ambiguity that accompanied social unrest which can easily trigger effects to other aspects of life (33). As such, it is reasonable to find anxiety even among those who are not actively engaged in social unrest.

Similar to the anxiety findings, social unrest related distress according to the engagement level revealed the same trend. Participants who oppose the law reform whether their engagement is high or low reported higher distress while those who support the law reform showed the lowest distress in both groups—high and low. Once again non engaging participants reported higher levels of social unrest related distress in comparison to their counterparts who support the law reform. These findings echoed the mental toll of social unrest and its harm to mental resilience of the entire society (2).

Differences in engagement in social unrest in reference to state-hope showed a complicated picture. That is, participants who support the law reform reported the highest level of state-hope, in both levels of engagement (low and high), while the lowest level of state-hope was reported among those against the law reform with low engagement. It might be that those who support the reform revealed the highest state-hope as most of them also voted for the current government in the most recent elections. As such, they look forward to applying the reform and expect to correct years of detrimental legal injustices that now may be remedied. Additionally, results show that participants who identified as not engaged in social unrest reported higher state-hope in comparison to their counterparts who opposed the law reform with low levels of engagement. However, among participants who oppose the law reform, state-hope was highest among those with high engagement compared to those with low engagement. A possible explanation for the differences in state-hope among those who oppose the reform may be that being active in both petitions and civil demonstrations (namely high engagement) provide more meaning in life because such activism may prevent the reform from coming to fruition. Indeed, previous studies stressed positive mutual association between hope (34) and state-hope (35) with meaning in life.

Finally, in reference to the predictors of mental indicators, our findings revealed that the main predictor that accounted for the explained variance of anxiety and social unrest related to distress was engagement in social unrest. As mentioned above, this finding supports previous studies such as Wray-Lake and colleagues (36) which found that high-cost political behaviors, such as attending a protest, predicted more depressive symptoms over time among young adults in a national U.S. sample. With regard to the demographic variables, distress related to social unrest was predicted by biological sex (female), religiosity (secular), and self-rated health (reported good or excellent). That is, female sex, those who are secular and those with higher self-rated health showed social unrest related distress. As for anxiety, it was predicted by age (older), biological sex (female), economic state (higher), and self-rated health (reported good or excellent). That is, older participants, female sex, those with higher economic status and those with higher self-rated health reported on anxiety. Indeed, demographic predictors were previously emphasized in relation to collective actions as variables that are associated with mental indicators such as post-traumatic stress disorder (PTSD) anxiety, and depression (2, 3). Specifically, Ni et al. (3) in their systematic review of collective actions and mental health, found that female sex and lower socioeconomic status were associated with increased risk of PTSD and anxiety symptoms while younger age correlated with anxiety symptoms. Moreover, Hou et al. (28) found in Hong Kong collective actions, that lower education levels and lower income levels were associated with greater odds of probable depression and suicidal ideation. Ni et al. (37) found that older individuals, who had less formal education, and had lower household income were overrepresented in the persistent moderate depression trajectory. In the present study, our findings revealed opposite results concerning the association between economic status and anxiety. This may be explained by the characteristics of the Israeli participants who are active in the present social unrest, and who mostly belong to the upper deciles of society (e.g., Hi-tech individuals, academics, physicians, lawyers, freelancers etc.). Moreover, according to the conservation of resources (COR) theory (38) resource loss is the central mechanism driving adaptation to stress. Thus, it might be that participants with higher economic status foresaw a threat of resource loss including abolishment of the rule of law and loss of places of employment.

With respect to the predictors of state-hope, results showed that demographic variables account for the higher contribution to the explained variance of state-hope. That is, older participants, those who are in a committed relationship, those with high levels of education, those with higher economic status and those with higher self-rated health demonstrated state-hope. Interestingly, the contribution of engagement in social unrest to the explained variance of state-hope was quite small. It might be that regardless of engagement in a collective action, state-hope during social unrest events is mostly associated with personal resources such as health, marital status, education and economic status which seem to be important especially during times of uncertainty which characterize periods of social unrest. Future replication studies with a longitudinal design are warranted to confirm the findings of this study.

### Limitations

Several limitations of this study should be noted. First, this study was conducted using a cross-sectional method, thus precluding any causal relationships among the variables. As such, longitudinal studies are warranted. Moreover, mental health indicators such as anxiety and psychological distress were not assessed prior to the present research. Thus, caution should be applied in interpreting the data. Second, the social unrest engagement measure used in the present study might not be able to capture the vast differences in the quality and the types of civic experiences Israeli people experienced following social unrest. Future research using a more encompassing measure of engagement in social unrest would add further evidence to these research findings. Third, as with any self-report study, response bias and social desirability could influence respondents' answers and discount data validity. This is especially salient regarding the social unrest distress measure. Future studies may consider a combination of methods that include valid instrument questionnaires and in-depth interviews to reduce response bias. Last, social unrest may differ according to the contest in which such engagement occurs. Thus, future studies are warranted to explore and evaluate the generalizability of the results to other nations in which their social unrest relates to legal reform that might affect national administration.

### Implications

Our results point toward the need for health-care and social work professionals to be aware of possible psychiatric sequelae during and after widespread civil unrest, not only among those who are politically active in the social unrest, but also among non-engaged participants who can also be affected as social unrest has the potential to affect the entire population. Moreover, health policy makers should plan services and short-term interventions to better protect public mental health during and following social unrest, especially among vulnerable subgroups such as those discussed in the present study.

## In conclusion

Social unrest is rising globally, and as such it is an important line of inquiry. The present study offers additional evidence for the association between social unrest and mental health in a sufficient representative population sample in Israel. In particular, our findings suggest that widespread social unrest could have a stronger adverse impact on the mental health of those who are engaged in social unrest (whether they oppose or support proposed legislation), but this mental toll may differ according to the level of the engagement and activism. Moreover, our results also highlight the mental burden of those who are not engaged in social unrest, as revealed in their reports regarding anxiety and distress. Surprisingly, state-hope seems to have a weak association with engagement in social unrest. Demographic variables such as economic status, self-rated health, education, age, marital status, and gender play a role in prediction of mental indicators and state-hope during social unrest. Concern about unmet mental health needs, during and following social unrest, should become an important focus for mental health professionals as well as for health policy makers.

## Data availability statement

The raw data supporting the conclusions of this article will be made available by the authors, without undue reservation.

## Ethics statement

The studies involving humans were approved by Ariel University Ethics Committee. The studies were conducted in accordance with the local legislation and institutional requirements. The participants provided their written informed consent to participate in this study.

## Author contributions

YH-R: Conceptualization, Data curation, Formal analysis, Investigation, Methodology, Project administration, Resources, Supervision, Validation, Visualization, Writing – original draft, Writing – review & editing. EL: Conceptualization, Data curation, Formal analysis, Funding acquisition, Investigation, Methodology, Resources, Validation, Visualization, Writing – original draft, Writing – review & editing. MB-E: Conceptualization, Data curation, Formal analysis, Investigation, Methodology, Project administration, Resources, Supervision, Validation, Visualization, Writing – original draft, Writing – review & editing.
